# PIEZO Channels in Mechano-Inflammation: Gatekeepers of Neuroimmune Crosstalk

**DOI:** 10.3390/diseases13080263

**Published:** 2025-08-15

**Authors:** Carmelo Pirri

**Affiliations:** Department of Neurosciences, Institute of Human Anatomy, University of Padova, 35121 Padova, Italy; carmelo.pirri@unipd.it

**Keywords:** PIEZO1, PIEZO2, mechano-inflammation, neuroimmune crosstalk, fibrosis, mechanotransduction, glia, macrophages, pain, PIEZO channels, fibroblasts

## Abstract

Mechanical forces shape immune responses in both health and disease. PIEZO1 and PIEZO2, two mechanosensitive ion channels, have emerged as critical transducers of these forces, influencing inflammation, pain, fibrosis, and neuroimmune regulation. This review aims to synthesize the current evidence on the role of PIEZO channels in mechano-inflammation, with a specific focus on their regulatory function in neuroimmune crosstalk. A comprehensive narrative synthesis was performed using the literature from PubMed, Scopus, and Web of Science up to June 2025. Experimental, translational, and mechanistic studies involving PIEZO channels in inflammatory, fibrotic, and neuroimmune processes were included. PIEZO1 is broadly expressed in immune cells, fibroblasts, and endothelial cells, where it regulates calcium-dependent activation of pro-inflammatory pathways, such as NF-kB and STAT1. PIEZO2, enriched in sensory neurons, contributes to mechanosensory amplification of inflammatory pain. Both channels are mechanistically involved in neuroinflammation, glial activation, blood–brain barrier dysfunction, connective tissue fibrosis, and visceral hypersensitivity. PIEZO channels act as integrators of biomechanical and immunological signaling. Their roles as context-dependent gatekeepers of neuroimmune crosstalk make them attractive targets for novel therapies.

## 1. Introduction

In recent years, the concept of mechano-inflammation, the mechanosensitive regulation of immune and inflammatory pathways, has emerged as a critical frontier in neuroscience, immunology, and regenerative medicine [1]. Central to this paradigm are PIEZO channels, a family of mechanically activated ion channels that sense physical deformation of the cellular membrane and transduce it into intracellular calcium signals. PIEZO1 and PIEZO2, first characterized in 2010, have since been recognized as essential regulators of tissue homeostasis, inflammation, and cellular mechanoadaptation [2].

Of particular relevance to the immune system is PIEZO1, which is expressed in various immune cells, including microglia, macrophages, dendritic cells, and lymphocytes [3]. These channels are activated by changes in extracellular matrix stiffness, cyclic stretch, shear stress, and hydrostatic pressure, mechanical features that are commonly altered in pathological states such as neurodegeneration, tumor progression, and fibrosis. Beyond their roles as mechanotransducers, PIEZO channels have emerged as central nodes in neuroimmune crosstalk, the bidirectional communication between nervous and immune systems mediated by cellular and molecular signaling [1,2,3]. Through their strategic localization in both neuronal and immune cell populations, PIEZO1 and PIEZO2 translate mechanical stress into coordinated neuroinflammatory responses, bridging peripheral and central immune regulation [1,2,3]. Experimental studies have demonstrated that PIEZO1 activation in microglia under hyperglycemic conditions induces pro-inflammatory signaling via JNK1 and mTOR, enhancing proliferation, migration, and phagocytic activity [1]. Similarly, in mechanically stressed fibroblasts from the human enthesis, PIEZO1 mediates neutrophil chemotaxis through calcium influx and upregulation of CXCL8, establishing a direct link between mechanical loading and acute inflammation [4]. Mechanistically, PIEZO1 serves as a gatekeeper of calcium-dependent immune pathways, including the NFAT1 transcriptional axis and interleukin signaling cascades [4,5]. PIEZO1-driven Ca^2+^ influx can influence not only inflammatory gene expression but also cytoskeletal remodeling, cell–cell interactions, and metabolic reprogramming, all of which are hallmarks of immune cell activation and migration [4,5]. In the central nervous system, PIEZO1 is increasingly implicated in the interface between neuronal microenvironments and resident immune cells. Tumor models, such as glioblastoma, show that PIEZO1 overexpression enhances cellular invasion and immunosuppressive signaling via YAP/TAZ activation and MAPK-mediated cytokine release [4,5]. These findings position PIEZO1 as a mechanosensitive orchestrator of the tumor immune microenvironment, with potential implications for both tumor progression and therapeutic resistance. Moreover, evidence suggests that PIEZO channels may act as context-sensitive modulators, capable of switching between pro- and anti-inflammatory roles depending on the cell type, mechanical milieu, and disease stage [1,2,3,4,5,6,7]. This duality highlights the therapeutic complexity but also the opportunity for precision in using physical or chemical modulators of PIEZO to rebalance dysregulated immune responses. Despite the growing number of individual studies, the field still lacks a systematic synthesis that evaluates the strength, quality, and translational relevance of the evidence. The present review addresses this gap by analyzing a curated set of studies focused on PIEZO channels and inflammation. By connecting the dots between mechanical forces, ion channel activity, and immune signaling, this review aims to define the merging field of mechano-inflammation through the lens of PIEZO biology.

## 2. Structural and Functional Overview of PIEZO Channels

PIEZO1 and PIEZO2 are large, non-selective mechanosensitive cation channels embedded in the plasma membrane, originally identified through a high-throughput screen in neuroblastoma cells [8]. Structurally, both channels form homotrimeric complexes with a propeller-like configuration, consisting of 38 transmembrane helices per monomer and a central ion-conducting pore. This unique architecture enables PIEZO proteins to function as highly sensitive mechanotransducers, directly gated by membrane tension without accessory proteins [8]. Functionally, PIEZO1 and PIEZO2 respond to diverse mechanical stimuli, such as membrane stretch, hydrostatic pressure, substrate stiffness, and shear stress. Upon activation, they allow the influx of cations, especially calcium, which acts as a second messenger in signaling cascades involved in inflammation, proliferation, migration, and differentiation. This process activates multiple intracellular pathways, including MAPK, NF-kb, PI3K-Akt, and RhoA/ROCK [9]. PIEZO1 is widely expressed in non-excitable cells such as vascular endothelial cells, renal epithelial cells, pulmonary alveolar cells, immune cells (e.g., macrophages and dendritic cells), and fibroblasts. In contrast, PIEZO2 is predominantly localized in sensory neurons, including dorsal root ganglia, trigeminal neurons, and Merkel cells, where it is critical for tactile sensation and proprioception [10]. Recent transcriptomic analyses have confirmed PIEZO1 upregulation in inflamed or fibrotic tissues and its induction by inflammatory cytokines IL-6 and transforming growth factor β (TGF-β) [9,10]. From a functional perspective, PIEZO1 plays key roles in regulating vascular tone, lymphatic valve development, and immune cell activation, while PIEZO2 governs mechanonociception, bladder fullness sensing, and lung inflation detection [1,10,11]. According to Selezneva et al. [12], PIEZO1-mediated calcium influx in immune cells modulates cytokine release and migratory behavior, while PIEZO2 controls sensory neuron excitation thresholds and neurogenic inflammation. Crucially, both PIEZO1 and PIEZO2 are subject to post-translational modifications and lipid interactions that modulate their mechanosensitivity. Cholesterol, PIP2, and cytoskeletal anchoring are known to influence channel kinetics and desensitization. Moreover, cross-regulation by mechanosensitive pathways, such as integrins and caveolins, fine-tunes PIEZO channel gating [13]. In addition to the force-from-lipids model, which describes the direct activation of PIEZO channels in response to changes in lipid bilayer tension, a second theory, known as the force-from-filaments model, has been proposed. In this model, the mechanosensitivity of PIEZO1 is regulated by physical interactions with the intracellular cytoskeleton (actin filaments, microtubules, and intermediate filaments) and with extracellular matrix (ECM) proteins. External mechanical forces are transmitted to the channels not only through membrane deformation but also via molecular “tethering” mediated by adhesion complexes such as integrins and linker proteins, including filamin, talin, and spectrin [14]. Experimental studies have shown that actin depolymerization or disruption of integrin–ECM interactions reduces PIEZO1 activity, whereas strengthening cytoskeleton–membrane connections can enhance its gating sensitivity [14,15]. This suggests that PIEZO1 acts as an integrated transducer of mechanical signals arising from both the lipid bilayer and cytoskeletal–extracellular scaffold [16]. For PIEZO2, however, the contribution of cytoskeletal and ECM proteins to gating regulation remains unclear and warrants further investigation. A growing body of evidence indicates that PIEZO1 is essential for transducing pathophysiological mechanical cues into pro-inflammatory outputs, in particular, in high-load or stiffened tissues, such as fibrotic lung or osteoarthritic cartilage. Conversely, PIEZO2 is central in encoding tactile and painful mechanical stimuli and may contribute to sensitization in chronic pain syndromes [17,18]. Thus, the distinct biophysical properties, distribution patterns, and signaling outcomes of PIEZO1 and PIEZO2 provide a mechanistic basis for their involvement in mechano-inflammation across different tissues and pathologies (Table 1).

## 3. PIEZO1 in Macrophage-Mediated Inflammatory Responses

Macrophages express functional PIEZO1 channels that respond to variants in matrix stiffness, osmotic pressure, hydrostatic pressure, and inflammatory cytokines. PIEZO1 serves as a mechanosensory transducer that influences innate immune functions through calcium signaling and downstream transcriptional reprogramming. Zhou et al. [19], using a model of human enthesis-derived fibroblasts co-cultured with peripheral immune cells, demonstrated that PIEZO1 activation via controlled cyclic mechanical stretch enhances neutrophil recruitment via NFAT1-dependent LIF expression. This supports the existence of a mechanically induced paracrine inflammatory axis within fibro-inflammatory niches [19,33]. Selezneva et al. [12] emphasized the contribution of PIEZO1 to monocyte-to-macrophage differentiation, cell polarization, and directional migration. Mechanistically, PIEZO1 activation induces rapid Ca^2+^ influx, promoting the activation of pro-inflammatory transcription factors including STAT1, NF-kB, and IRF5, all critical for M1 macrophage polarization. Zhang et al. [33] confirmed these effects in murine macrophages exposed to a fibrotic extracellular matrix (ECM), where PIEZO1 activity increased IL-6 and tumor necrosis factor α (TNF-α) transcription. Recent studies also highlighted PIEZO1’s role in the metabolic reprogramming of macrophages [20,34].

Savadipour et al. observed that PIEZO1-dependent calcium influx supports a metabolic switch toward aerobic glycolysis, enhancing mitochondrial ROS generation, hallmarks of activated inflammatory macrophages [13]. Furthermore, PIEZO1 expression itself is induced by matrix stiffness and mechanical tension, generating a feedforward loop that reinforces inflammatory activation in fibrotic or stiffened tissues [35]. In addition to its role in tissue homeostasis and inflammation, PIEZO1 has been implicated in pathological remodeling. PIEZO1 activation promotes monocyte infiltration into inflamed vascular walls and fibrotic organs, contributing to chronic inflammation and tissue damage. Wang et al. demonstrated that pharmacological inhibition of PIEZO1 using GsMTx4 or siRNA silencing markedly reduced cytokine secretion, dampened macrophage recruitment, and favored M2 phenotype induction with elevated expression of IL-10 and arginase-1 [36]. These findings position PIEZO1 as a mechanosensitive gatekeeper of macrophage-driven inflammation. Its regulatory role spans from immune surveillance to pathological fibrosis and tumor-associated macrophage activation, making it a promising target in the context of diseases such as pulmonary fibrosis, atherosclerosis, rheumatoid arthritis, and solid tumors [21].

## 4. PIEZO in Neuroinflammation and Glial Activation

**Figure 1:**
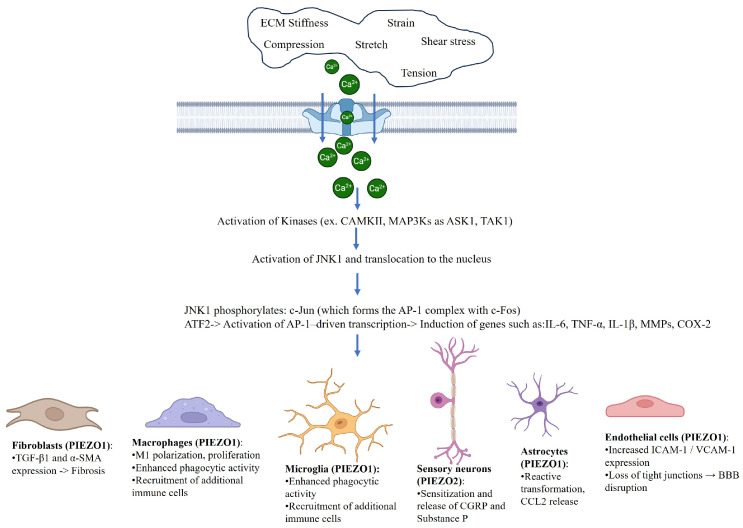
Mechanosensitive regulation of inflammatory crosstalk by PIEZO channels. Created in Biorender. Carmelo Pirri. (2025) https://BioRender.com/3urnk5h.

Microglial cells and astrocytes, the primary innate immune effectors in the central nervous system (CNS), express PIEZO1 and respond dynamically to mechanical stimuli and metabolic stressors. Liu et al. [22], using BV2 microglial cells subjected to acute hyperglycemia, demonstrated that PIEZO1 activation triggers the JNK1 and mTOR signaling pathways, resulting in enhanced proliferation, migration, and the release of pro-inflammatory cytokines such as IL-1β. Zheng et al. [23] showed that substrate stiffness, mimicking the rigid microenvironments of inflamed or fibrotic brain regions, upregulates PIEZO1 expression in primary microglia and leads to NF-kB activation, with subsequent production of IL-6 and TNF-α. PIEZO1 also contributes to the acquisition of the ameboid, pro-inflammatory microglial phenotype, suggesting a mechanosensitive basis for microglial activation. Beyond microglia, PIEZO1 has been shown to affect astrocytic behavior [23]. Procès et al. [29] described that astrocytes exposed to mechanical strain exhibit PIEZO1-dependent calcium signaling that regulates their polarity, migration, and inflammatory gene expression, including CCL2 and IL-1β. These alterations influence the formation of glial scars and the progression of neurodegeneration. Emerging in vivo data further suggest that PIEZO1 may regulate neuroimmune interactions at the level of the blood–brain barrier and neuronal–glial synapses. Zhong et al. [10] linked PIEZO1 activity to vascular inflammation and leukocyte infiltration into the CNS parenchyma. In addition, PIEZO1 has been associated with altered microglial response in neurodegenerative conditions such as Alzheimer’s disease and multiple sclerosis, where it may modulate chronic inflammation, phagocytic clearance of debris, and neuronal survival [23]. Overall, these findings position PIEZO1 as a crucial transducer of mechanical and metabolic cues within the CNS, contributing to glial activation and neuroinflammatory pathogenesis. Targeting PIEZO1 in glial cells may represent a novel therapeutic strategy to mitigate neuroinflammation and limit disease progression in a range of CNS disorders (Figure 1).

### Dual Role of PIEZO Channels in Inflammation: A Context-Sensitive Paradigm

One of the most intriguing features of PIEZO channel activity is its context-sensitive duality. While PIEZO1 and PIEZO2 are often implicated in pro-inflammatory signaling, amplifying cytokine release, immune cell recruitment, and tissue remodeling, they can also mediate anti-inflammatory or homeostatic effects depending on the mechanical environment, cellular phenotype, and disease stage [5,10,17]. For instance, PIEZO1 activation in endothelial cells may support vascular integrity and immune surveillance under physiological shear stress, while the same channel promotes pathological leukocyte extravasation and cytokine overproduction under ischemic or pro-inflammatory conditions. In the same way, PIEZO2, known for enhancing mechanical pain under inflammatory sensitization, contributes to protective mechanosensation in the intact peripheral nervous system. This bidirectional capability reinforces PIEZO channels as nuanced regulators rather than linear effectors, with therapeutic implications requiring precision and timing [5,10,17].

## 5. PIEZO2: Pain Sensation and Sensory Neuroinflammation

PIEZO2 is a key mechanosensitive ion channel expressed, in particular, in sensory neurons and has been shown to be essential for tactile transduction, proprioception, and the development of mechanical allodynia. Its role in neurogenic inflammation and pain modulation is increasingly supported by both experimental and clinical evidence. Jo et al. [17] demonstrated in murine models of inflammatory and neuropathic pain that pharmacological blockade of PIEZO2 significantly reduces mechanical hyperalgesia and tactile allodynia. This effect was associated with reduced activation of spinal dorsal horn neurons and decreased expression of pain-related neuropeptides [30]. The results support the idea that PIEZO2 contributes to peripheral sensitization and spinal amplification of pain signals [30]. Giniatullin et al. [18] further explored PIEZO2’s interaction with transient receptor potential (TRP) channels. In particular, TRPV1 and acid-sensing ion channels (ASICs) show that PIEZO2 can potentiate nociceptive signaling via synergistic crosstalk, especially under conditions of inflammation and tissue acidosis. This interaction may be relevant in chronic pain conditions, such as migraine and neuropathic pain [22,37]. In migraine models, PIEZO1 and PIEZO2 were found to be co-expressed in trigeminal afferents, where they contribute to the mechanosensory amplification of vascular pulsation-induced pain [38]. PIEZO2’s involvement in mechanical headache pain is also supported by studies showing its increased expression in dural afferents during inflammation. In addition, in models of post-traumatic pain and peripheral nerve injury, PIEZO2 contributes to the hyperexcitability of mechanonociceptors, suggesting a role in the persistence of pain beyond tissue recovery [13]. Sonkodi et al. [31] described PIEZO2 as a central gatekeeper in the mechanosensory–neuroimmune interface. PIEZO2 activation alters the sensory neuron threshold and neurotransmitter release, facilitating the propagation of inflammatory signals to the spinal cord. Knockout studies in mice confirm that deletion of PIEZO2 in nociceptors impairs behavioral responses to mechanical pain stimuli while preserving thermal sensitivity, indicating modality-specific involvement [39]. PIEZO2 expression is modulated by inflammatory cytokines such as IL-6 and NGF, further linking its activity to the inflammatory milieu. In the gastrointestinal system, PIEZO2 has also been implicated in visceral hypersensitivity, as observed in models of irritable bowel syndrome and bladder pain syndrome, where mechanical stretch of visceral afferents leads to PIEZO2-dependent firing and release of neuropeptides like CGRP and substance P [32]. Altogether, PIEZO2 serves as both a sensory detector and amplifier of noxious mechanical inputs, linking tissue mechanics to neuroimmune pain circuits. Its modulation presents a promising therapeutic avenue in the treatment of chronic pain syndromes, mechanical hypersensitivity, migraine, and visceral pain disorders [32,39].

## 6. PIEZO1 and Vascular Inflammation in the Central Nervous System

Cerebral endothelial cells express high levels of PIEZO1, which functions as a critical mechanosensor for shear stress and a modulator of inflammatory responses. Upon activation by hemodynamic forces or pro-inflammatory cytokines, PIEZO1 regulates endothelial calcium influx, nitric oxide (NO) production, cytoskeletal dynamics, and tight-junction integrity [10]. Under physiological conditions, PIEZO1 contributes to cerebral autoregulation, modulates vascular tone via calcium-dependent eNOS activation, and maintains barrier function through the regulation of actin stress fibers and junctional protein expression. However, during pathological conditions such as inflammation, stroke, or traumatic brain injury, PIEZO1 becomes maladaptive [10]. Zhong et al. showed that PIEZO1 enhances the expression of the adhesion molecules ICAM-1 and VCAM-1 in endothelial cells exposed to inflammatory stimuli, promoting leukocyte adhesion and diapedesis across the blood–brain barrier (BBB). This is a key initiating event in neuroinflammation. In addition, PIEZO1 activation leads to increased actin stress fiber formation and RhoA/ROCK pathway activation, weakening tight junctions and promoting vascular leakage [10,40]. In ischemia–reperfusion models, PIEZO1-mediated calcium overload has been associated with mitochondrial dysfunction, ROS generation, and subsequent upregulation of pro-inflammatory mediators, including IL-1β, IL-6, and MCP-1 [10,41]. These molecular events culminate in endothelial activation, BBB disruption, and secondary neurotoxicity. In the context of glioblastoma and CNS tumors, PIEZO1 is often overexpressed [24,40,42]. Cieśluk et al. [25] demonstrated that in glioma cells, PIEZO1 promotes vascular mimicry, upregulation of VEGF, and the expression of matrix metalloproteinases (MMP-2 and MMP-9), facilitating tumor invasion and immune evasion. This reinforces the dual inflammatory and proangiogenic role of PIEZO1 in the CNS microenvironment. Furthermore, PIEZO1 has been implicated in age-related cerebrovascular dysfunction [25]. Li et al. [26] hypothesized that PIEZO1 contributes to vascular cognitive impairment by altering neurovascular coupling and mechanosensitive tone regulation. Chronic PIEZO1 hyperactivation could impair perfusion stability and accelerate neurodegenerative progression, in particular, in the context of hypertension or arterial stiffness [26].

These findings underscore the dualistic nature of PIEZO1 signaling in the CNS vasculature: it is beneficial under physiological conditions but deleterious when dysregulated [27,28]. In the future, pharmacological PIEZO1 blockade or biomechanical modulation may preserve BBB integrity, attenuate leukocyte infiltration, and limit neurovascular inflammation in disorders such as multiple sclerosis, vascular dementia, and CNS malignancies.

## 7. PIEZO and Connective Tissue: Mechanotransduction and Fibrotic Regulation

In connective tissues, PIEZO1 serves as a central mechanosensitive transducer that governs fibroblast behavior, extracellular matrix (ECM) remodeling, and the development of fibrosis. Mechanical forces such as matrix stiffness, stretch, and compression are sensed through PIEZO1, leading to calcium influx and the activation of fibrotic transcriptional programs. He et al. [43] demonstrated that increased matrix stiffness directly activates PIEZO1 in dermal fibroblasts, resulting in enhanced TGF-β1 expression and increased collagen I and fibronectin deposition. This PIEZO1-mediated signaling cascade also includes Smad2/3 phosphorylation and activation of pro-fibrotic transcriptional programs. These findings are supported by in vivo models showing that PIEZO1 contributes to wound contraction and dermal scar thickening in response to mechanical load [43,44]. Some studies [43,44,45,46,47] provided further insight, showing that PIEZO1 activates the YAP/TAZ pathway in human fibroblasts and tenocytes, facilitating their differentiation into contractile, α-SMA-expressing myofibroblasts. This transdifferentiation is a key event in chronic fibrotic disease progression, including tendinopathy, systemic sclerosis, and keloid formation [43,44,45,46,47]. YAP/TAZ signaling, activated downstream of PIEZO1, also modulates expression of CTGF and integrins, further amplifying the fibrotic response. Zhou et al. [4] confirmed that PIEZO1 activation in human enthesis fibroblasts subjected to mechanical stretch induces secretion of leukemia inhibitory factor (LIF) and drives neutrophil chemotaxis. These findings suggest a dual role of PIEZO1 in both fibrosis and immune cell recruitment, reinforcing its centrality in mechano-inflammation. PIEZO1 links mechanical overload to sustained fibroblast activation and secretion of pro-inflammatory cytokines such as IL-6, IL-8, and CCL2. This mechanosensitive inflammatory axis contributes not only to fibrosis but also to persistent low-grade inflammation in mechanically stressed tissues [43,44,45,46,47]. In addition, PIEZO1 interacts with other mechanoreceptors, such as integrins and TRVP4, creating a cooperative signaling network that fine-tunes cellular responses to mechanical stresses. In lung fibroblasts, PIEZO1 activation under cyclical stretch conditions induces the expression of pro-fibrotic genes and promotes fibroblast-to-myofibroblast transition [48]. In the same way, in liver stellate cells and renal pericytes, increased PIEZO1 activity correlates with fibrogenic remodeling and loss of tissue compliance [49,50]. Furthermore, PIEZO1 expression is dynamically regulated by the extracellular environment: fibrotic tissue exhibited increased PIEZO1 mRNA and protein levels, creating a feed-forward loop between mechanical stress and fibrotic activation. Inhibiting PIEZO1 in vitro and in vivo has been shown to reduce fibroblast contractility, lower ECM gene expression, and prevent tissue stiffening [43,44,45,46,47].

## 8. Clinical and Translational Perspectives

The translational relevance of PIEZO channels spans multiple disease contexts, linking mechanotransduction to chronic inflammation, tissue degeneration, fibrosis, and pain. In osteoarthritis models, Steinecker-Frohnwieser et al. [51] demonstrated that PIEZO1 activation in human chondrocytes exposed to cyclic mechanical stretch and IL-1β results in the upregulation of IL-6, MMP13, and markers of apoptosis. Treatment with the PIEZO1 antagonist GsMTx4 prevented these effects, preserving chondrocyte viability and reducing catabolic signaling, thus offering a potential disease-modifying strategy [51]. Brylka et al. [52] demonstrated that in osteoarthritis, elevated PIEZO1 expression in chondrocytes increases mechanosensitive calcium influx, which, in turn, activates catabolic pathways, promoting ECM degradation and accelerating cartilage degeneration. In pulmonary settings, Migulina et al. [48] identified PIEZO1 as a central driver of ventilator-induced lung injury and fibrosis. Under cyclic stretch, PIEZO1 activation in lung macrophages led to increased HIF1α signaling and endothelin-1 production, exacerbating fibro-inflammatory remodeling. Genetic or pharmacological inhibition of PIEZO1 attenuated these effects, highlighting its potential as a therapeutic target in critical care and pulmonary fibrosis [44]. Metabolic inflammation also involves PIEZO1, as shown by Zhao et al. [53], who reported that adipocyte-specific PIEZO1 activation in obesogenic conditions enhanced IL-6 and leptin secretion, contributing to systemic insulin resistance (Table 2).

These findings underscore the link between mechanical strain in expanding adipose tissue and chronic low-grade inflammation, further implicating PIEZO1 in metabolic syndrome [54]. Beyond these organ-specific contexts, PIEZO channels have been implicated in several emerging areas. In migraine models, PIEZO2 contributes to trigeminal hypersensitivity, while PIEZO1 regulates dural endothelial permeability, offering new entry points for treating neurovascular headache syndromes [55]. In oncology, PIEZO1 promotes tumor angiogenesis and immune evasion through VEGF and MMP upregulation [55,56]. In the gut, PIEZO1 and PIEZO2 are involved in serotonin release from enterochromaffin cells, potentially modulating gut–brain axis inflammation [23]. Recent developments include ultrasound-induced PIEZO inhibition in gastrointestinal afferents to reduce visceral hypersensitivity and bioengineered scaffolds designed to deliver targeted mechanical cues that suppress PIEZO-driven fibrotic or inflammatory pathways [57].

Future translational research should prioritize the development of selective modulators, establish delivery platforms for local mechano-modulation, and conduct clinical trials to validate PIEZO-targeted strategies in human disease.

## 9. Discussion: PIEZO Channels as Converging Hubs of Neuroimmune Mechanotransduction

The emerging role of PIEZO channels as mechanosensitive regulators of inflammation, immunity, and neurobiology underscores a paradigm shift in our understanding of tissue pathophysiology. Far beyond their classical functions in touch and proprioception, PIEZO1 and PIEZO2 operate as polymodal sensors that integrate biomechanical stress with intracellular signaling, thereby orchestrating immune activation, glial reactivity, vascular inflammation, and fibrotic remodeling [8,9,10,11,12,13,14,15,16,17,18,19,20,21,33,34,35,36]. This mechanistic convergence is evident in neuroimmune interfaces, where PIEZO1-mediated calcium influx serves as a master regulator of both pro-inflammatory transcriptional programs and cytoskeletal dynamics. Microglia and macrophages respond to changes in matrix stiffness or metabolic cues via PIEZO1 activation, triggering cascades that culminate in cytokine release, phagocytic priming, and polarization [12,18,23,30,35,37]. Similarly, PIEZO2 participates in the sensory encoding of mechanical pain while amplifying neurogenic inflammation in chronic pain conditions and visceral hypersensitivity. A recurring theme across systems is the context-dependent duality of PIEZO activity: it is beneficial for homeostasis under physiological loading, yet deleterious when hyperactivated in disease states such as fibrosis, neurodegeneration, or cancer. This Janus-faced behavior of PIEZO channels positions them as critical “gatekeepers” of neuroimmune crosstalk, fine-tuning the interface between mechanical forces and immune dynamics across the CNS, connective tissue, and peripheral organs. The spatial and temporal regulation of PIEZO channels appears tightly interwoven with their interaction partners, YAP/TAZ, integrins, TRP channels, and inflammatory mediators, creating a layered regulatory architecture that is both complex and targetable. These characteristics make PIEZO proteins highly attractive for future therapies and treatments to modulate them [58,59]. Despite the mechanistic advances highlighted in this review, critical gaps remain. The absence of standardized models for assessing PIEZO function under physiological versus pathological load, limited data from human tissues, and the lack of highly selective PIEZO modulators constrain current translational applications. Moreover, the functional interplay between PIEZO1 and PIEZO2, in particular, in tissues where both isoforms are co-expressed, remains incompletely understood. The neuroimmune implications of PIEZO channels remain a fertile ground for discovery, with potential ramifications for treating diverse conditions.

## 10. Conclusions

PIEZO channels have emerged as pivotal mechanosensory transducers that translate physical forces into immune and inflammatory signaling, thereby shaping cellular responses in health and disease. Their expression across immune cells, glia, sensory neurons and fibroblasts situates them at the heart of mechano-inflammation, where mechanical and immunological domains intersect. As “gatekeepers of neuroimmune crosstalk”, PIEZO1 and PIEZO2 modulate a spectrum of processes, including macrophage activation, glial inflammation, sensory hypersensitivity, and fibrotic remodeling. Their mechanistic versatility and widespread distribution render them promising yet complex targets for therapeutic modulation. The integration of PIEZO biology into translational medicine will create a new class of interventions that restore immune and mechanical homeostasis by recalibrating the mechanosensitive dialogue between cells and their microenvironment.

## Figures and Tables

**Table 1:** PIEZO channels: cell-type specific expression, mechanical stimuli, and mechano-inflammatory outcomes.

Cell Type	PIEZO Isoform	Mechanical Stimulus	Functional Outcome	Key References
Macrophages	PIEZO1	Matrix stiffness and stretch	M1 polarization and IL-6/TNF-α release	[12,19,20]
Microglia	PIEZO1	Hyperglycemia and substrate stiffness	NF-kB activation and IL-1β production	[21,22]
Astrocytes	PIEZO1	Mechanical strain	Polarity, migration, and CCL2/IL-1β expression	[23]
Fibroblasts	PIEZO1	Tension and compression	Myofibroblast transition and TGF-β expression	[24,25,26,27,28]
Sensory neurons	PIEZO2	Tactile/mechanical load	Mechanical allodynia and neuropeptide release	[17,29,30,31]
Endothelial cells	PIEZO1	Shear stress and inflammation	ICAM/VCAM expression barrier breakdown	[10,32]

**Table 2:** PIEZO channels in disease-associated mechano-inflammation.

Disease Model	PIEZO Isoform	Tissue/Cell Target	Pathological Role	Possible Modulation Strategy	References
Osteoarthritis	PIEZO1	Chondrocytes	Promotes IL-6 and MMP13 expression under mechanical stretch + IL-1β; induces apoptosis	GsMTx4 antagonist protects cartilage integrity	[51,52]
Pulmonary fibrosis/VILI	PIEZO1	Lung macrophages and fibroblasts	Enhances HIF1α, endothelin-1, and fibrotic remodeling under cyclic stretch	Genetic silencing or pharmacological inhibition	[44,46]
Metabolic syndrome	PIEZO1	Adipocytes	Increases IL-6 and leptin secretion under obesogenic stress, promoting insulin resistance	Targeted inhibition reduces low-grade inflammation	[53]
Migraine and pain syndromes	PIEZO1 + PIEZO2	Trigeminal afferents and dural vasculature	PIEZO2 contributes to tactile hypersensitivity; PIEZO1 regulates endothelial permeability and inflammation	PIEZO2 inhibition reduces pain; PIEZO1 inhibition modulates BBB	[10,31,39]
Chronic neuroinflammation	PIEZO1	Microglia, astrocytes, and endothelial cells	Triggers JNK/mTOR and NF-kB signaling; promotes cytokine production, leucocyte recruitment, and BBB breakdown	siRNA or GsMTx4 inhibition dampens inflammatory signaling	[1,10,18,30,37]
Systemic sclerosis/fibrosis	PIEZO1	Fibroblast and myofibroblasts	Induces TGF-β1 and α-SMA expression; drives myofibroblast differentiation and collagen deposition	Inhibition reduces ECM gene expression and contractility	[4,46]
Tendinopathy/enthesitis	PIEZO1	Fibroblasts from enthesis	Promotes neutrophil recruitment via LIF under stretch; contributes to local inflammation	Mechanical unloading or PIEZO inhibition	[4,13,19,33]
Irritable bowel syndrome/bladder pain	PIEZO2	Visceral afferents	Mediates visceral hypersensitivity, CGRP, and substance P release under stretch	PIEZO2 antagonists reduce afferent firing and nociception	[31,39,40]
Glioblastoma/CNS tumors	PIEZO1	Glioma cells and CNS endothelium	Promotes VEGF, MMPs, and immune evasion; supports vascular mimicry and tumor progression	Pharmacological blockade reduces invasion and vascular remodeling	[10,24,40,41,42]
Vascular cognitive impairment/aging	PIEZO1	Cerebral endothelium	Alters neurovascular coupling and increases ROS and BBB permeability under chronic stress	Long-term PIEZO modulation under investigation	[25,26,27,28]

## Data Availability

The data presented in this study are available upon request from the author. The data are not publicly available due to privacy.

## Abbreviations

The following abbreviations were used in this manuscript:

NF-kB	Nuclear factor kappa-light-chain-enhancer of activated B cells
STAT1	Signal transducer and activator of transcription 1
JNK1	c-Jun N-terminal kinase 1
CXCL8	C-X-C motif chemokine ligand 8
NFAT1	Nuclear factor of activated T cells 1
ECM	Extracellular matrix
PI3K-Akt	Phosphatidylinositol 3-kinase—protein kinase B
IRF5	Interferon regulatory factor 5
CCL2	C-C motif chemokine ligand 2
MMP13	Matrix metalloproteinase-13
ICAM	Intercellular adhesion molecule
VCAM	Vascular cell adhesion molecule
